# An Atypical Case of Methemoglobinemia due to Self-Administered Benzocaine

**DOI:** 10.1155/2015/670979

**Published:** 2015-03-19

**Authors:** Thomas M. Nappe, Anthony M. Pacelli, Kenneth Katz

**Affiliations:** Department of Emergency Medicine, Lehigh Valley Hospital/USF Morsani College of Medicine, Cedar Crest Boulevard and Interstate 78, Allentown, PA 18103, USA

## Abstract

Acquired methemoglobinemia is an uncommon hemoglobinopathy that results from exposure to oxidizing agents, such as chemicals or medications. Although, as reported in the adult population, it happens most often due to prescribed medication or procedural anesthesia and not due to easily accessed over-the-counter medications, the authors will describe an otherwise healthy male adult with no known medical history and no prescribed medications, who presented to the emergency department reporting generalized weakness, shortness of breath, headache, dizziness, and pale gray skin. In addition, the patient reported that he also had a severe toothache for several days, which he had been self-treating with an over-the-counter oral benzocaine gel. Ultimately, the diagnosis of methemoglobinemia was made by clinical history, physical examination, and the appearance of chocolate-colored blood and arterial blood gas (ABG) with cooximetry. After 2 mg/kg of intravenous methylene blue was administered, the patient had complete resolution of all signs and symptoms. This case illustrates that emergency physicians should be keenly aware of the potential of toxic hemoglobinopathy secondary to over-the-counter, nonprescribed medications. Discussion with patients regarding the dangers of inappropriate use of these medicines is imperative, as such warnings are typically not evident on product labels.

## 1. Introduction

Acquired methemoglobinemia is typically caused by oxidative stress and many prescribed medications are strongly associated with inducing methemoglobinemia (Table 1) [1, 2]. A very common presentation of this cyanotic illness is after a medical procedure, such as endoscopy or bronchoscopy, during which a liberal amount of local anesthetic, such as benzocaine spray, is used [1–3]. However, one very rarely develops methemoglobinemia from self-administering an over-the-counter medication.

It is quite unusual for a normal, healthy adult to acquire methemoglobinemia from self-administered, oral benzocaine gel. A literature review uncovered only two cases of over-the-counter benzocaine gel-related induction of methemoglobinemia. In a 2004 retrospective study of 198 adverse reactions to benzocaine reported to the FDA, only one of 132 adults with methemoglobinemia was reported to have developed it as a result of using benzocaine gel [3]. The second case was a six-year-old child, reported in 2010, whose toothache was treated with 7.5% benzocaine gel (Baby Orajel) [4]. In fact, it is so rare for methemoglobinemia to be acquired in this fashion, that, in a 2013 study, with 576 participants, evaluating the efficacy of self-applied, over-the-counter oral benzocaine gel, there was no incidence of methemoglobinemia, even after a 1,026 mg administration by one participant in a two-hour period [5]. The reported maximum dose before inducing methemoglobinemia would be 15 mg per kilogram for a 50 kg person [5]. The authors report a rare case of an otherwise healthy adult patient who presents with methemoglobinemia after self-administering over-the-counter topical benzocaine gel. This unique case, along with a brief description of acquired methemoglobinemia and its presentation, diagnosis, and treatment, is described.

## 2. Case

**Figure 1:**
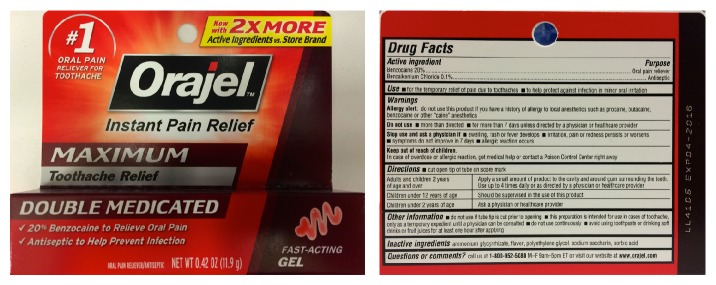
Orajel with ingredients including benzocaine 20%.

A 29-year-old male of Chinese descent with no known medical history and no prescribed medications presented to the emergency department with a chief complaint of generalized weakness since the previous evening. He also reported dyspnea, headache, and dizziness, which started the day of presentation, and his coworkers noted his skin to be pale and grayish in appearance. The patient also reported that he had a toothache for several days and was self-treating with an over-the-counter topical medication, Maximum Strength Orajel (benzocaine) (Figure 1). He stated he had been applying the gel three times per day for three days.

**Figure 2:**
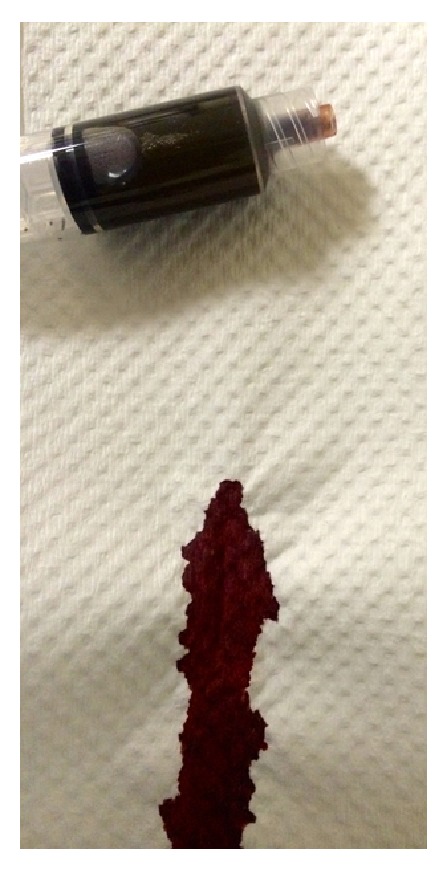
Chocolate-brown blood.

Physical examination revealed an alert, mildly distressed, cyanotic-appearing man. His vital signs revealed a temperature of 98.7°F, heart rate of 70 beats per minute, blood pressure of 145/68 mmHg, respirations at 16 breaths per minute, and an oxygen saturation (SpO_2_) of 88% on six liters per minute oxygen via nasal cannula. The patient's skin displayed moderate pallor with perioral cyanosis. Upon initial venous blood draw, his blood had an abnormal chocolate-brown appearance (Figure 2).

Laboratory studies revealed an unremarkable complete blood count and comprehensive metabolic profile. An arterial blood gas with cooximetry measured pH, 7.32 (7.35–7.45); pCO_2_, 42 mm Hg (35–48); pO_2_, 178.0 mm Hg (83–108); HCO_3_−, 24.3 mEq/L (21–28); SaO_2_, 99% (95–98); total calculated hemoglobin, 17.3 g/dL; oxyhemoglobin, 71.9% (95.0–98.0); carboxyhemoglobin, 0.0% (0.5–1–5); methemoglobin, 27.4% (<3.0) with an oxygen content of 17.8 mL/dL (15.0–33.0).

The diagnosis of methemoglobinemia was made in conjunction with consultation with a medical toxicologist and 2 mg/kg intravenous methylene blue was administered. His symptoms improved within fifteen minutes and he felt markedly better within an hour. Repeat cooximetry measured his oxyhemoglobin at 94.5% and methemoglobin at 0.9%. After a four-hour stay in the emergency department and complete resolution of his signs and symptoms, he was discharged with the instruction to discontinue the use of Orajel and seek appropriate dental care.

## 3. Discussion

Methemoglobinemia is a hemoglobinopathy that can be either inherited or acquired [1–3, 6]. Acquired methemoglobinemia is due to the exposure to an oxidizing chemical or drug (Table 1), leading to the removal of an electron from ferrous hemoglobin (Fe^2+^) to create ferric hemoglobin (Fe^3+^) at a rate that surpasses the endogenous reducing mechanisms, which primarily include the enzymatic activity of cytochrome b5 reductase and nicotinamide adenine dinucleotide (NADH) methemoglobin reductase [1–3, 6]. The resultant ferric hemoglobin does not release oxygen to target tissue, causing a leftward shift of the oxygen dissociation curve, leading to a functional anemia that can progress to cyanosis and even death [1–3, 6].

The classic presentation of a patient with methemoglobinemia is dyspnea, pallor, grayish skin, cyanosis, and hypoxia, which does not improve with supplemental oxygen administration [1, 2]. The clinical manifestation of methemoglobinemia is directly correlated to the level of measured methemoglobin, and symptoms can be worsened by extremes of age and comorbidities that may alter the levels of preexisting normal hemoglobin (Table 2) [1–3].

Diagnosis of methemoglobinemia is made by the patient's clinical presentation, with the presence of refractory hypoxemia and chocolate-colored blood and is confirmed by an arterial blood gas with cooximetry [1–3, 7, 8]. Cooximetry provides a method of differentiating between various states of hemoglobin, including measurements of total hemoglobin, oxyhemoglobin, deoxyhemoglobin, methemoglobin, carboxyhemoglobin, and sulfhemoglobin [1, 7, 8]. As present in this case, an oxygen saturation (SaO_2_) gap is evident on ABG when compared to pulse oximetry (SpO_2_). This means that the ABG displays a falsely normal oxygen saturation (SaO_2_), since this is a* calculation* based on the measured serum partial pressure of oxygen (PO_2_), given the assumption that all the patient's hemoglobin is normal (i.e., oxy- or deoxyhemoglobin); thus, the methemoglobin is included in the percentage of saturated hemoglobin in the resulting, overestimated SaO_2_ [7, 8]. This is in contrast to pulse oximetry (SpO_2_), which is a* measurement* of the wavelengths of oxyhemoglobin and deoxyhemoglobin, with oxyhemoglobin given as a percentage of total hemoglobin [7, 8]. Therefore, cooximetry is used as a more direct method of measurement for confirmatory testing [1, 2, 7, 8].

Treatment with oxygen and the antidote, methylene blue (1-2 mg/kg of 1% solution intravenously over five minutes), is indicated for symptomatic patients or methemoglobin levels greater than 25 to 30% [1, 2, 9]. Methylene blue accelerates the reduction of methemoglobin to hemoglobin by stimulating the activity of the nicotinamide adenine dinucleotide phosphate (NADPH) methemoglobin reductase, an enzyme that ordinarily plays a very little role in the normal reduction of methemoglobin [1]. Utilizing NADPH from the hexose monophosphate shunt, NADPH methemoglobin reductase then reduces methylene blue to leukomethylene blue, which then donates an electron to reduce methemoglobin (Fe^3+^) back to hemoglobin (Fe^2+^) [1, 2]. After the administration of methylene blue, improvement should be seen in minutes; otherwise, a second dose can be given within 30 to 60 minutes [1, 2]. If no improvement takes place after the second dose, other contributing factors or etiologies may be present, such as G6PD deficiency or the presence of long-acting oxidizing agents, and exchange transfusion may be considered [1].

## 4. Conclusion

Acquired methemoglobinemia is a toxic hemoglobinopathy commonly caused by prescribed medications or those administered in a hospital setting. Rarely, however, over-the counter, self-administered medications containing benzocaine can cause methemoglobinemia in otherwise healthy adults. In this setting, emergency physicians should be keenly aware of this potentially life-threatening condition, its diagnosis and treatment, and consultation with a medical toxicologist is recommended in all cases.

## Figures and Tables

**Table 1:** Drugs known to induce methemoglobinemia (not inclusive) [1, 2].

Amyl nitrite Benzocaine Bupivacaine Dapsone Lidocaine Metoclopramide	Nitric oxide Nitroglycerin Nitroprusside Nitrofuran Phenazopyridine	Prilocaine Quinones (e.g., chloroquine) Rifampin Sulfonamides (e.g., sulfamethoxazole)

**Table 2:** The clinical manifestation of methemoglobinemia [1, 2].

%MetHgb	Symptomology
0–3	Asymptomatic and normal level in blood
3–15	Possibly asymptomatic, grayish pallor, mild cyanosis, and low oxygen saturation per pulse oximeter
15–20	Cyanosis and chocolate-brown appearance of blood
20–50	Dyspnea, fatigue, weakness, headache, dizziness, and syncope
50–70	CNS depression, lethargy, seizures, and metabolic acidosis
>70	Severe cyanosis and death
